# Coordinated plant and microbial transcriptional responses to oil-sands process–affected water

**DOI:** 10.1093/ismeco/ycag152

**Published:** 2026-06-01

**Authors:** Julius E Nweze, Simon Morvan, Abdul Samad, Marie-Josée Bergeron, Dani Degenhardt, Julien Tremblay, Kyle Symonds, Douglas G Muench, Christine Martineau, Etienne Yergeau

**Affiliations:** Centre Armand Frappier Santé et Biotechnologie, Institut National de Recherche Scientifique, Laval, Quebec, H7V 1B7, Canada; Centre Armand Frappier Santé et Biotechnologie, Institut National de Recherche Scientifique, Laval, Quebec, H7V 1B7, Canada; Centre Armand Frappier Santé et Biotechnologie, Institut National de Recherche Scientifique, Laval, Quebec, H7V 1B7, Canada; Laurentian Forestry Centre, Canadian Forest Service, Natural Resources Canada, Québec, Quebec, G1V 4C7, Canada; Northern Forestry Centre, Canadian Forest Service, Natural Resources Canada, Edmonton, Alberta, T6H 3S5, Canada; Centre Armand Frappier Santé et Biotechnologie, Institut National de Recherche Scientifique, Laval, Quebec, H7V 1B7, Canada; Department of Biological Sciences, University of Calgary, Calgary, Alberta, T2N 1N4, Canada; Department of Biological Sciences, University of Calgary, Calgary, Alberta, T2N 1N4, Canada; Laurentian Forestry Centre, Canadian Forest Service, Natural Resources Canada, Québec, Quebec, G1V 4C7, Canada; Centre Armand Frappier Santé et Biotechnologie, Institut National de Recherche Scientifique, Laval, Quebec, H7V 1B7, Canada

**Keywords:** oil-sands process–affected water, naphthenic acid fraction compounds, metatranscriptomics, constructed wetland systems, root microbiome, xenobiotic detoxification

## Abstract

Constructed wetland treatment systems (CWTSs) are promising options for treating oil-sands process–affected water (OSPW), which contains toxic naphthenic acid fraction compounds (NAFCs). However, the molecular mechanisms underlying NAFCs attenuation by plants and root microbes remain poorly resolved. In our previous mesocosm study, *Typha latifolia* increased NAFC removal 2.5-fold relative to unplanted controls without reducing plant growth. Here, using RNA from that same experimental system, we applied metatranscriptomics to 40 root samples collected over 60 days to characterize plant and active microbial responses to OSPW exposure. The active root-associated microbial community was dominated by *Pseudomonadota*, and *Burkholderiales* remained the most active order, although *Flavobacteriaceae* (*Bacteroidota*) activity increased with time when exposed to OSPW. Microbial community composition shifted with both time and water type, and 42 genes with potential roles in NAFC or related organic-compound transformation were differentially expressed in OSPW mesocosms. These responses were dominated by oxidoreductases affiliated mainly with *Burkholderiales* and *Rhizobiales*. The host plant also responded strongly to OSPW, up-regulating genes encoding oxidoreductases, transporters, and glycosyltransferases associated with xenobiotic stress and detoxification. Together, these results revealed coordinated plant and microbial transcriptional responses in a system where enhanced NAFC attenuation had already been demonstrated chemically. The observed patterns, however, likely reflect the broader OSPW mixture rather than NAFCs alone.

## Introduction

Oil-sands process–affected water (OSPW), generated during the hot-water extraction of bitumen, contains a complex mixture of dissolved organic and inorganic compounds, with naphthenic acid fraction compound (NAFC) representing one of the major toxic pollutants [1]. NAFCs comprise classical naphthenic acids, together with naturally occurring dissolved organic matter and other bitumen-derived carboxylic acids [2]. Many proposed OSPW treatments remain energy-intensive, costly, or dependent on *ex situ* processing [3], which has motivated interest in sustainable in situ alternatives such as plant- and microbe-based systems. Recent work in constructed wetland treatment systems (CWTSs) has begun to define both treatment performance and the microbial pathways or genomic potential associated with NAFC attenuation in OSPW-exposed systems [4–7]. Related rhizosphere studies in constructed wetland and phytoremediation settings further suggest that root-associated microbial communities are likely to be important contributors to contaminant transformation [8–10]. However, the actively expressed microbial functions and plant responses operating in intact wetland root systems remain poorly understood.

Microorganisms are central to bioremediation because of their remarkable ability to break down complex organic pollutants through a wide range of enzymatic processes, ultimately leading to degradation, detoxification, or immobilization [11]. Both bacteria and fungi can metabolize toxic hydrocarbons under aerobic or anaerobic conditions, using them as sources of carbon and energy and producing less harmful by-products in the process [12]. This ability is linked to a range of enzyme systems, including oxidoreductases (e.g. dehydrogenases, cytochrome P450s), hydrolases, laccases, dehalogenases, lipases, and proteases, involved in the breakdown of hydrocarbons in various environments [13]. Although microbial degradation of NAFCs and NAFC-like compounds has been reported [4], only a few NAFC-degradation microbes, genes, and pathways have been identified.

Candidate aerobic NAFC degradation pathways include beta-oxidation (ring cleavage) with α-oxidation, ω-oxidation, aromatization, or CoA-thioester activation, whereas proposed anaerobic routes include benzoyl-CoA-linked processes coupled to nitrate, sulfate, or iron-reducing metabolism, or methanogens [4]. Even so, most studies have emphasized community composition in tailings ponds or wetland systems using DGGE, 16S ribosomal RNA (rRNA) gene profiling, metagenomics, or enrichment approaches [4, 14, 15], leaving a gap between inferred metabolic potential and the genes actively expressed during exposure in plant-associated environments.

Plants complement microbial degradation through uptake, detoxification, immobilization, stabilization, or degradation of diverse organic pollutants [16]. In CWTSs, roots can stimulate microbial activity by releasing exudates, supplying oxygen, and reshaping rhizosphere habitat [8, 9, 17]. Wetland plants such as *Typha* spp. can also accumulate and transform organic contaminants while supporting diverse root-associated microbiomes [16, 18]. By contrast, the plant transcriptomic response to NAFC-containing OSPW remains much less characterized than the microbial side, particularly in whole root systems [19].

In our previous greenhouse mesocosm study, *Typha latifolia* significantly reduced total NAFCs relative to unplanted controls and altered water and substrate chemistry [20]. Here, we use RNA from that same experimental system to provide the mechanistic transcriptomic context for those previously measured treatment outcomes. This study addresses two unresolved questions: which members of the root-associated community are transcriptionally active during OSPW exposure, and whether the host plant mounts a detoxification-related transcriptional response in the same mesocosms. Specifically, we compared root metatranscriptomes from OSPW- and reverse-osmosis water (ROW)–filled mesocosms over 60 days to identify active taxa and differentially expressed plant and microbial genes associated with exposure to OSPW.

## Materials and methods

### Study site, materials, plant propagation, and mesocosm setup

The source of coarse sand tailings (CSTs) and OSPW, propagation of *T. latifolia*, and the full greenhouse mesocosm design were described previously in Balaberda *et al*. [20]. Briefly, the experiment was conducted under controlled greenhouse conditions to minimize environmental variability. *Typha latifolia* was propagated from field-collected seeds (zone DM1.3; dry mixedwood) for 3 months in peat-filled styroblock containers before transplanting. Twelve plants were transplanted into each mesocosm (average height 120.8 ± 17.0 cm). Each mesocosm contained a 20 cm layer of CST (85.6 L) and either OSPW or ROW (106.9 L) maintained 25 cm above the substrate. For the metatranscriptomic component, we used a 2 × 2 factorial design with four replicates per treatment (*n* = 16): ROW without plants, ROW planted with *T. latifolia*, OSPW without plants, and OSPW planted with *T. latifolia*. After a ROW acclimation period, the mesocosms were flushed for 24 h and then refilled with the assigned water type to begin the 60-day exposure period. Root sampling was destructive and performed on Days 0 (twice: before and after exposure to OSPW or refilling with ROW), 6, 19, 40, and 60, yielding 40 root samples for sequencing.

Both CST and OSPW were chemically characterized in the paired mesocosm study from which the RNA for the current study was obtained [20]. CST consisted predominantly of sand (98.0% ± 0.0), with minor silt and clay fractions. Relative to ROW, OSPW was characterized by higher salinity, alkalinity, and major ion concentrations typical of tailings pond water, including elevated Ca, Na, K, Cl, and SO_4_. In that same study, planted mesocosms altered water chemistry through reductions in salinity, alkalinity, and selected ions over time. Those measurements provide the chemical context for interpreting the transcriptional responses reported here.

### Root sampling and RNA extraction

Root samples were collected destructively immediately after the ROW acclimation period and 24-h flush, before the mesocosms were refilled with their assigned water type (D0) and 6, 19, 40, and 60 days after refilling with either ROW or OSPW (D6, D19, D40, and D60). Roots were immediately cleaned after sampling, snap-frozen in a dry ice-ethanol bath, shipped on dry ice, and stored at −80^∘^C. Prior to RNA isolation, frozen root tissues (150–200 mg) were ground using 5 mm stainless-steel beads and a TissueLyser II (Qiagen). RNA was extracted using the NucleoSpin RNA plant and fungi kit (Macherey-Nagel). ERCC ExFold RNA Spike-In Mix 1 (Invitrogen) was used as internal control and mixed together with PFL and PFR buffers. Two DNase treatments were performed, using the TURBO DNA-free kit (Invitrogen). Concentrated RNA extracts were recovered using the RNA Clean & Concentrator-25 kit (Zymo Research). Absence of residual DNA in the RNA extracts was confirmed by 16S PCR [primers 515F-Y: 5′-GTGYCAGCMGCCGCGGTAA [21] and 926R: 5′-CCGYCAATTYMTTTRAGTTT [22]. RNA quality and quantity were assessed using the 2100 Bioanalyzer (Agilent) and Qubit 3 (Invitrogen) instruments, respectively.

### Library preparation and sequencing

rRNA was depleted from 2 μg of root RNA using the Pan-Plant riboPOOL kit (siTOOLs Biotech) and streptavidin-coated magnetic beads (New England Biolabs). Size-selective purification and concentration were performed using the SPRIselect bead-based reagent (Beckman Coulter) and the RNA Clean & Concentrator-5 kit (Zymo Research), respectively. RNA was eluted in 10 μl of water and quantified using the Qubit RNA High Sensitivity assay kit (Invitrogen). Libraries were prepared using the NEBNext Ultra II RNA Library Prep kit for Illumina (New England Biolabs). Based on the RNA insert size, fragmentation incubation time was set to 8 min at 94°C. NEBNext Multiplex Oligos were used for the PCR enrichment of adaptor-ligated DNA step and the number of PCR cycles was set to eight, based on the rRNA-depleted RNA input amount. Library size and concentration were assessed using the High Sensitivity DNA kit (Agilent) and 2100 Bioanalyzer and the Qubit dsDNA High Sensitivity assay kit (Invitrogen), respectively. Equimolar libraries (3 ng/μl) were pooled and sequenced at the Centre d’expertise et de services, Génome Québec (Montréal, Canada), using a 25B flow cell and the NovaSeq X Plus system (Illumina) for 2 × 100 cycles (paired-end mode). A phiX library was used as a control and mixed at 1% level.

### Microbial community and beta diversity

The microbial community in metatranscriptomic raw reads was profiled using SingleM v0.18.3 [23], which targets active 35 bacterial and 37 archaeal single-copy marker genes. The resulting operational taxonomic unit (OTU) table was transformed into a matrix in R. Alpha diversity (Observed richness and Shannon index) was calculated on raw counts to assess community complexity over water type and time. We fitted linear mixed-effects models with water type, time, and their interaction as fixed effects and included mesocosm as a random intercept to account for repeated sampling within the experiment. Beta diversity was evaluated using Bray–Curtis dissimilarity matrices calculated from relative abundance-transformed data and visualized via Principal Coordinates Analysis (PCoA). Formal multivariate testing was conducted using PERMANOVA (adonis2, 999 permutations) with the model Bray-Curtis ~ water type * time. Beta-dispersion was also tested with betadisper and permutation tests for both water type alone and the combined water-type-by-time grouping structure. Within SingleM, hierarchical clustering was performed using the unweighted pair group method with arithmetic mean (UPGMA) algorithm with jackknife support values [24]. The taxon-based dissimilarity between the samples across the time points was generated, and it ranges from 0.0 (low dissimilarity) to 1.0 (high dissimilarity).

### Metatranscriptomic read processing and assembly

Sequences were processed using an in-house bioinformatics pipeline [25] built on the GenPipes workflow [26]. Reads were quality-controlled using Trimmomatic v0.39 [27] and BBDUK (BBTools v38.1), and co-assembled using MEGAHIT v1.2.9 [28]. The quality-check (QC)-passed reads were mapped with BWA mem v0.7.17 [29] against contigs to assess the quality of the assembly. Reads were then mapped using STAR v2.7.11b [30] against the *T. latifolia* reference genome (National Center for Biotechnology Information (NCBI): JAAWWQ000000000.1) to separate plant (mapped) from nonplant/microbial (unmapped) transcripts.

### Differential gene expression analysis

Transcript abundances were analysed using DESeq2 [31] to identify differentially expressed genes (DEGs) in cattail roots between OSPW and ROW at the same five time points (0, 6, 19, 40, and 60 days). Here, Day 0 represents samples collected immediately after draining the ROW at the end of the acclimation period, before exposure to OSPW or fresh ROW treatments. Before running the analysis, only genes with ≥10 counts in ≥2 replicates were retained. We used Benjamini–Hochberg [32] correction to control the false discovery rate (FDR), and genes were considered differentially expressed if the adjusted *P*-value was ≤.05 and the log2-fold-change (log_2_​FC) was ≥1.

### Gene prediction and assignment

Gene prediction used Prodigal v2.6.3 [33], and predicted genes were assigned to KEGG orthologs (KOs) using HMMER v3.3.2 [34] against the KOFAM database [35] with a significance threshold of 10^−10^. Targeted homology searches for NAFC-degradation genes (e-value 1e^−5^) included: (i) 18 previously associated genes (e-value of 1e^−5^; [3]) and (ii) hydrocarbon degradation markers (cutoffs: ≥25% coverage, ≥30% identity, e-value ≤1e^−5^) using the Calgary approach to ANnoTating HYDrocarbon degradation genes (CANT-HYD) database containing 37 HMMs [36] and the Hydrocarbon Aerobic Degradation Enzymes and Genes (HADEG) database containing 259 proteins [37]. Functional profiling for biogeochemical cycles employed curated databases for methane (MCycDB), nitrogen (NcycDB), sulfur (SCycDB), and phosphorus (PCycDB). The Basic Local Alignment Search Tool (BLAST) identity thresholds were ≥75% for McycDB [38], NcycDB [39], and ScycDB [40] cyclings and 30% for PCycDB cycling [41]. Gene abundance profiles were generated by mapping QC-passed reads against predicted genes using BWA mem v0.7.17 [29]. Taxonomic assignment used BLAST (e-value = 10^−10^) against NCBI databases [42]. All the generated data were visualized in R [43], using ggplot2 [44] and Interactive Tree of Life (iTOL) [45]. The generated scalable vector graphics (SVG) were annotated in Inkscape and exported as Portable Network Graphics (PNG).

## Results and discussion

### Experimental baseline and naphthenic acid fraction compound removal

**Figure 1:**
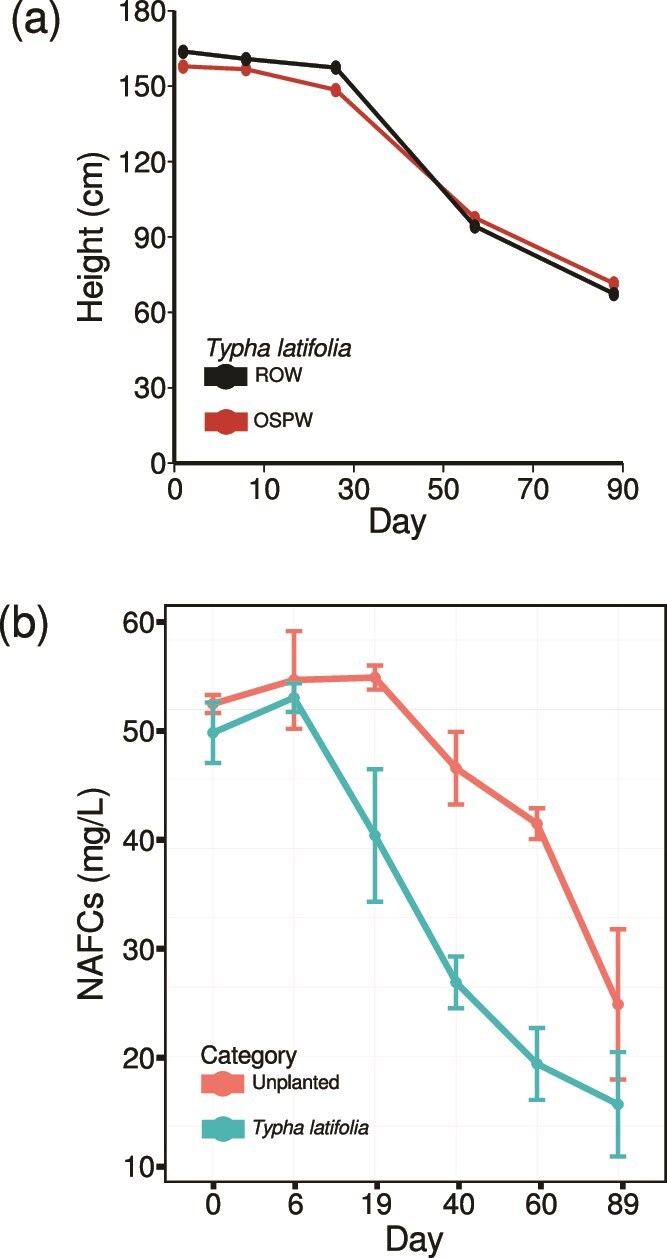
Plant performance and NAFC removal in the paired mesocosm study from which RNA was obtained for the present metatranscriptomic analysis. (a) *Typha latifolia* height over time in mesocosms supplied with oil-sands process–affected water (OSPW) and reverse osmosis water (ROW). (b) Temporal change in mean NAFC concentration in planted and unplanted OSPW mesocosms. The paired study reported overall NAFC removals of 40% in planted mesocosms and 16% in unplanted controls [20]. Times 0–60 represent sampling days after adding OSPW or refilling with ROW. This figure is reproduced from Balaberda *et al*. [20] to provide the chemical and plant-performance context for the transcriptomic analyses presented here.

As context for the metatranscriptomic analysis, we first considered the plant performance and chemical outcomes previously measured in this same mesocosm experiment. *Typha latifolia* maintained >95% survival in both OSPW and reverse osmosis water (ROW) [20], confirming its resilience under the conditions used here [46]. Although some shoots showed 70%–99% necrotic tissue, that visual damage was attributed in the paired study to an uncontrolled aphid infestation rather than to a significant OSPW effect on plant height, biomass, or overall vigor (*P* > .05; Fig. 1a). Panel 1b shows the temporal change in NAFC concentration in the paired OSPW mesocosms; in that study, these trajectories corresponded to overall NAFC removals of 40% in planted mesocosms and 16% in unplanted controls [20]. Plant presence also influenced salinity, alkalinity, and ion concentrations in the system. We use that previously quantified attenuation as the functional backdrop for the metatranscriptomic patterns described below.

### De novo assembly information of the root microbial metatranscriptome

RNA was extracted and sequenced from 40 *T. latifolia* root samples collected from OSPW and ROW (Table S1; Table S2A). After quality-filtering and coassembly, the reads yielded 17 541 contigs (Table S2B). Contig lengths ranged from 1000 to 16 907 (bp); 21 contigs exceeded 10 kb, and the assembly N50 was 1604 bp.

### Active microbial community in the root samples

**Figure 2:**
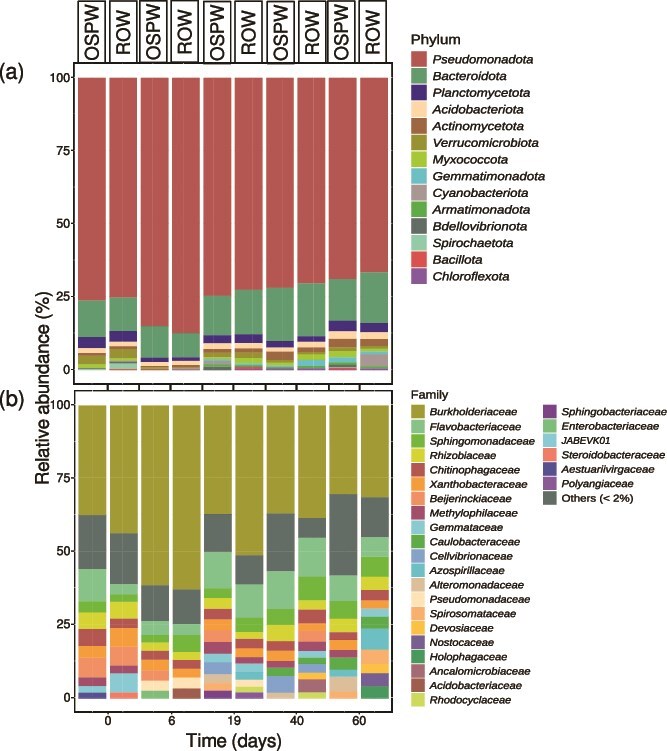
Active microbial profile of root-associated *T. latifolia* in mesocosms containing oil-sands process–affected water (OSPW) or reverse osmosis water (ROW). (a) Phylum-level relative abundance. (b) Family-level relative abundance. Profiles were generated from metatranscriptomic reads using expressed universal-single-copy genes. Time 0 is before addition of OSPW; 6–60 represent sampling days after adding OSPW or refilling with ROW.

We examined the composition and activity of the root-associated microbial community over time. Metatranscriptome profiling of root-associated prokaryotic communities in both OSPW and ROW mesocosms showed a clear dominance of *Pseudomonadota* across all time points (Fig. 2a; Table S3 and Table S4A), peaking on D6 at 85% in OSPW and 87% in ROW after draining and refilling. After D6, *Pseudomonadota* declined but remained more abundant in OSPW than in ROW. As *Pseudomonadota* abundance decreased, *Bacteroidota* increased. In OSPW samples, active *Bacteroidota* ranged from 11% to 18%, whereas in ROW it ranged from 8% to 18%.

*Bacteroidota* abundance increased steadily from D6 to D40 in both water types before decreasing at D60. *Planctomycetota* was the third most abundant phylum in OSPW samples, ranging from 1% to 4%, except on D40, where *Actinomycetota* showed a slight increase at 1%. In our DNA-based study [20], while *Pseudomonadota* and *Bacteroidota* were likewise the most abundant phyla detected in *T. latifolia* roots, whereas *Actinomycetota* and *Verrucomicrobiota* were more abundant than *Planctomycetota.* This contrast suggests that *Planctomycetota* may be less abundant overall yet comparatively more transcriptionally active in roots exposed to OSPW.

In the ROW samples, different phyla occupied the third position in relative abundance over time, including *Planctomycetota, Acidobacteriota, Gemmatimonadota*, and *Cyanobacteriota*. The dominance of *Pseudomonadota* in active *T. latifolia* root communities is notable because many members of this phylum are associated with the metabolism of polycyclic aromatic hydrocarbons and other complex organic contaminants [47], and their dominance in active *T. latifolia* root bacterial communities further supports the relevance of this plant species in remediation systems. However, in the present study this pattern should be interpreted as evidence of a community well suited to chemically complex conditions, rather than as direct proof of NAFC degradation. The trend is consistent with other *Typha*-planted tailings environments [48], although it differs from some natural wetlands, where *Actinobacteria, Bacillota, Pseudomonadota*, and *Planctomycetota* often co-dominate the root zone [49]*.*

At the family level, *Burkholderiaceae* was consistently the most active family at all time points in both water types (Fig. 2b; Table S3 and Table S4B), rising sharply at D6 (61% in OSPW; 63% in ROW) after draining and refilling, and then declining toward D60 (30% in OSPW; 31% in ROW). Relative abundance was higher in ROW than OSPW because other families, especially *Flavobacteriaceae* (*Bacteroidota*), contributed more strongly to OSPW samples. In the paired DNA-based study of *T. latifolia* roots, *Burkholderiaceae* was also dominant, followed by *Chitinophagaceae, Xanthobacteraceae,* and *Sphingomonadaceae;* by contrast, the roots of another aquatic plant, *Juncus balticus*, were dominated by *Gallionellaceae* [20]. The prominence of *Burkholderiaceae* is consistent with reports linking this family to tolerance of heavy metals and hydrocarbon contaminants [50, 51]. Several *Burkholderia* lineages are also well known as plant growth-promoting rhizobacteria (PGPR) that enhance plant resilience under stressful conditions [52]. *Flavobacteriaceae,* which are often associated with plant health, growth, and tolerance to abiotic stress [53], was the second most abundant family in OSPW (5%–13%) and D19 (12%) and D40 (13%) in ROW, whereas other second-tier families, including *Beijerinckiaceae, Sphingomonadaceae Azospirillaceae,* and *Xanthobacteraceae*, varied by time point. These families are root-associated taxa that can support plant growth and resilience, including in *Typha* spp. [9, 54].

### Microbial diversity between samples at different times

**Figure 3:**
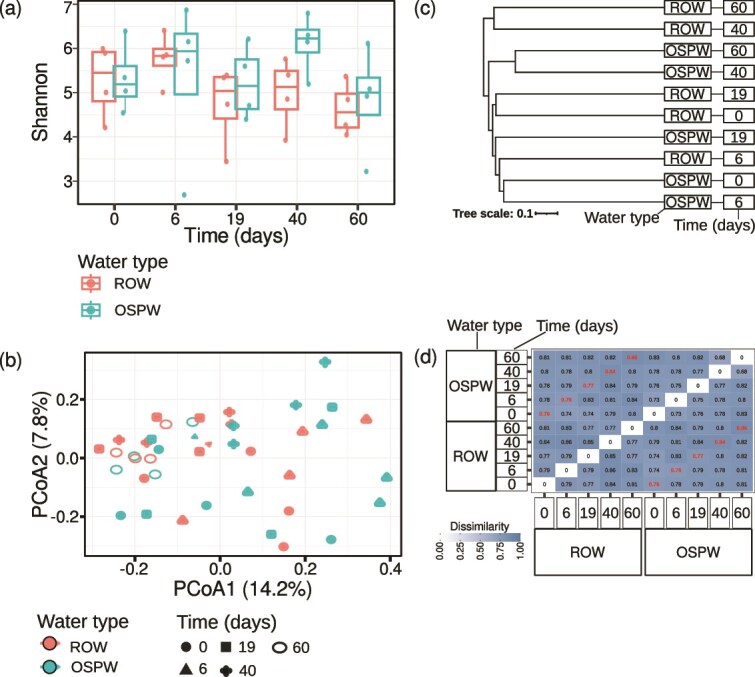
Diversity and dissimilarity of active microbial communities associated with the roots of *T. latifolia* in mesocosms containing OSPW or ROW. (a) Shannon diversity across water type and time, showing nonsignificant effects of water type, time, and their interaction. (b) Principal Coordinates Analysis (PCoA) based on Bray–Curtis dissimilarity illustrating community succession patterns. (c) Dendrogram showing sample clustering across time points. (d) Bray–Curtis dissimilarity values between water type at different time points based on the SingleM-derived taxonomic profiles. It ranges from 0.0 (low dissimilarity) to 1.0 (high dissimilarity). Time 0 indicates samples collected before OSPW or fresh ROW treatment period; times 6–60 represent sampling days after adding OSPW or refilling with ROW.

Alpha diversity was comparatively stable between water types (Fig. 3a; Table S5A). Observed richness was not significantly affected by water type (*F* = 1.51, *P* = .265), time (*F* = 1.09, *P* = .383), or their interaction (*F* = 0.90, *P* = .478). Shannon diversity was likewise not significantly affected by the water type (*F* = 0.76, *P* = .418), time (*F* = 1.18, *P* = .344), or their interaction (*F* = 0.73, *P* = .583). These results are consistent with the paired metagenomic analysis, which also showed no significant alpha-diversity differences between water types or across sampling times [20]. In contrast, beta diversity showed clear multivariate restructuring over the time course of the experiment (Fig. 3b and c; Table S5B). Bray–Curtis PCoA and hierarchical clustering both indicated separation by time and water type, and PERMANOVA supported an overall effect of the combined model (*R*^2^ = 0.293, *P* = .001). Beta-dispersion did not differ among the combined water-type-by-day groups (permutation test, *P* = .27), suggesting that the temporal-treatment pattern was not driven solely by differences in within-group dispersion. Samples from early and late time points formed distinct clusters, indicating that time was a major driver of community restructuring. As D0 samples were collected immediately after the acclimation phase and before the treatment period began, we interpret D0 primarily as a baseline reference rather than as a treatment-defined state. The pronounced shift at D6 likely reflects a combination of early biological response to the new water chemistry and system-level disturbance associated with draining and refilling. A similar temporal pattern was observed in our DNA-based study of *T. latifolia*, where D40, D60, and D89 samples clustered separately from the earlier times regardless of water type [20]. In the present metatranscriptomic dataset, Bray–Curtis dissimilarity ranged from 0.76 to 0.86 (Fig. 3b; Table S5), indicating substantial turnover in the active community over time. We therefore interpret the increasing divergence primarily as evidence of strong temporal restructuring, with OSPW contributing to an additional selective pressure. The relatively high dissimilarity already present at D0 likely reflects baseline root-to-root heterogeneity, sampling immediately after draining the acclimation water, RNA extraction, and technical variation inherent to marker-gene-based active community profiling.

### Plants’ differentially expressed genes and active detoxification

**Figure 4:**
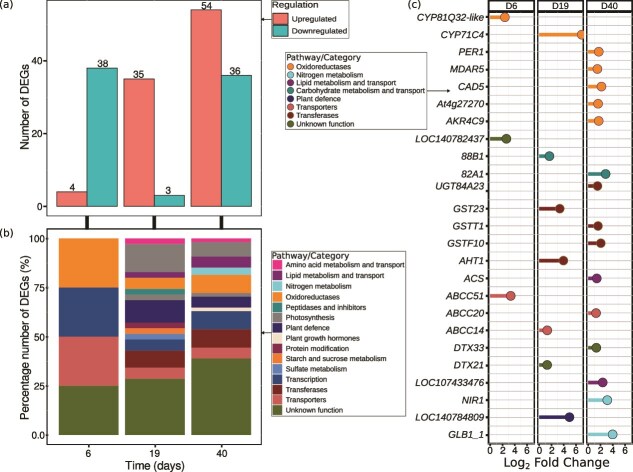
Differentially expressed plant genes (DEGs) from plant roots. (a) Number of DEGs identified through differential expression analysis in roots grown in mesocosms mimicking constructed wetland systems. (b) Functional categories of the DEGs. (c) Selected up-regulated genes identified in the analysis. Each time (0, 6, 19, 40, and 60) was compared between systems containing oil-sands process–affected water (OSPW) and reverse osmosis water (ROW). No DEGs were found on Day 60. Time 0 is before addition of OSPW; times 6–60 represent sampling days after adding OSPW or refilling with ROW.

Because planted OSPW mesocosms in the paired study removed more NAFCs than unplanted controls [20], we asked whether plant roots displayed transcriptional signatures consistent with xenobiotic stress response and detoxification. Genes responsive to NAFC exposure are expected to belong to broader detoxification pathways that process structurally complex xenobiotics, so we analyzed plant-derived DEGs to identify *T. latifolia* genes associated with chemically complex OSPW exposure (Fig. 4a; Table S6). Up-regulated genes were identified at D6 (*n* = 3), D19 (*n* = 35), and D40 (*n* = 54), suggesting that the host plant mounted an active molecular response rather than functioning solely as a passive substrate for microbes. These DEGs were mainly classified as oxidoreductases, transferases, carbohydrate transport, plant defense, and transporters (Fig. 4b; Table S7). At D19 and D40, dominant categories included carbohydrate metabolism, oxidoreductases, lipid metabolism and transport, plant defense, transcription, transferases, amino acid metabolism and transport, peptidases and inhibitors, and photosynthesis. As noted above, these responses likely reflect the broader dissolved mixture in OSPW rather than NAFCs alone.

We identified 3, 7, and 15 up-regulated plant genes at D6, D19, and D40, respectively, that could plausibly contribute to NAFC-related or broader xenobiotic transformation processes (Fig. 4c, Table S7). Most of these candidates were oxidoreductases. The responsive DEGs identified in *T. latifolia* were similar to transcripts reported previously in *Arabidopsis thaliana* and *Salix bebbiana* exposed to contaminant stress [19, 55]. Plant xenobiotics metabolism is often divided into three phases: transformation, conjugation, and compartmentalization/exudation [56]. The oxidoreductases identified here are consistent with a phase I–style detoxification response, although their direct substrates cannot be assigned from transcript data alone. Cytochrome p450s (CYP450s) were identified at the first two times (D6 and D19), where CYP81Q32-like and CYP71C4 were up-regulated, respectively (Fig. 4c). CYP450s catalyze the oxygenation of diverse xenobiotics in eukaryotes and are often thought to catalyze the first step in xenobiotic metabolism which correlates with our findings [57]. CYP71C4 catalyzes the selective oxygenation or epoxidation of indole derivatives to indoxyl (Phase I detoxification), a precursor of indole alkaloids and other compounds important for plant growth and defense [58, 59]. It can also result in indole degradation or unproductive indigo formation [60]. At D40, other oxidoreductases are up-regulated including, peroxidase (PER1), monodehydroascorbate reductase (MDAR5), cinnamyl alcohol dehydrogenase (*CAD5*), NAD(P)H dehydrogenase (quinone; *At4g27270*), and NADPH-dependent aldo-keto reductase (*AKR4C9*). *MDAR5* catalyzes the conversion of monodehydroascorbate to ascorbate while oxidizing nicotinamide adenine dinucleotide (NADH) and is known to mediate detoxification of environmental pollutants such as 2,4,6-trinitrotoluene and 1-chloro-2,4-dinitrobenzene [61].

Although alcohol dehydrogenases (*ADHs*) have been proposed to participate in degrading naphthenic acids and polycyclic aromatic compounds in microbes [62, 63], this function has not been documented in plants; however, the plant enzyme *CAD5*, which reduces aldehydes to alcohols, may possess similar catalytic potential [64, 65]. NAD(P)H-dependent oxidoreductases catalyze a wide variety of redox reactions with many different substrates [66]. *AKR4C9* also acts on a broad range of substrates, including reducing ketosteroids, aromatic aldehydes, ketones, other aliphatic aldehydes, and oxidizes hydroxysteroids [67].

The phase II metabolic encoding genes, uridine diphosphate (UDP) glycosyltransferases (UGTs), were identified at each time point. At D6, 19, and 40, we found that crocetin glucosyltransferase (*LOC140782437*), UDP-glycosyltransferase 88B1-like (*UGT88B1*), and UDP-glycosyltransferase 82A1 (*UGT82A1*) and gallate 1-beta-glucosyltransferase (*UGT84A23*) were up-regulated, respectively (Fig. 4c). The *UGT88B1* gene encodes a protein essential for phase II detoxification, glycosylating lipophilic endogenous and xenobiotic compounds [68]. Further, *UGT84A23* catalyzes the formation of 1-O-β-D-glucose esters from hydroxybenzoic acids and cinnamic acid derivatives [69]. The second most abundant phase II detoxification gene family was glutathione-S-transferases (GSTs) where *GST23* was upregulated at D19 and *GSTT1* and *GSTF10* were upregulated at D40 (Fig. 4c). Both UGTs and GSTs are common phase II detoxification enzymes that link sugars or glutathione to electrophilic functional groups on xenobiotics to increase their water solubility and facilitate transport [74]. GSTs also function in the detoxification of reactive oxygen species, which NAFCs have been reported to produce in plant and animal systems [75, 76]. Other up-regulated transferases were identified at D19, agmatine hydroxycinnamoyl transferase 1-like (*AHT1*) and D40, acetyl-coenzyme A synthetase (*ACS*). *AHT1* catalyzes the first step in antifungal hydroxycinnamoyl agmatine biosynthesis by transferring acyl groups from p-coumaroyl-CoA and feruloyl-CoA to acyl acceptors [77, 78]. Further, *ACS* has been reported in microbes as part of the degradation pathway for cyclohexene carboxylic acids and may also be involved in the beta-oxidation of some NAFCs [3].

Finally, the last group of genes up-regulated by OSPW were two transporter gene families, the ABC (ATP-binding cassette) transporter type C and the DETOXIFICATION or multidrug and toxic compound efflux carriers (*DTX/MATE*). At D6, 19, and 40 the ABCCs 51, 14, and 20 were up-regulated, respectively (Fig. 4c). ABCCs are involved in detoxification of organics and heavy metals, catabolite and phytohormone transport, and ion channel regulation [70]. The *DTX* transporters were less abundant but both *DTX21* and *DTX33* were up-regulated at D19 and D40, respectively (Fig. 4c). Similar to ABC transporters, *DTXs* have been shown to transport organic and inorganic compounds and although *DTX21* does not have a known function, *DTX33* has been shown to transport chloride ions in *Arabidopsis* and this *DTX* may not be involved with NAFC transport [71]. Transporter activity is notable, potentially facilitating NAFC uptake [72]. Other notable DEGs include, 14 kDa proline-rich protein DC2.15-like (*LOC107433476*), ferredoxin-nitrite reductase (*NIR1*), anaerobic nitrite reductase (*GLB1_1*). The proline-rich protein (*LOC107433476*) plays a role in maintaining the strong drought resistance of *Stipa purpurea* population [73]. *NIR1* and *GLB1_1* are involved in nitrate reduction and are noteworthy because nitrate-reducing processes have been implicated in anaerobic NAFC degradation in cooperation with methanogens and sulfate- or iron-reducing microbes [4].

Overall, the substantial up-regulation of detoxification-related genes in *T. latifolia*’s roots supports an active plant response to OSPW exposure. These responses may contribute to the transformation, conjugation, or compartmentalization of organic constituents and could alter the chemical environment experienced by root-associated microbes. However, the present data do not allow us to assign those responses specifically to NAFCs or to conclude that the plant directly prepares compounds for subsequent microbial degradation.

### Microbial differentially expressed genes and their taxonomic origins

The microbial DEG patterns are best interpreted in the context of the stronger NAFC attenuation previously observed in planted mesocosms [20]. In that paired study, planted systems showed lower NAFC concentrations than unplanted controls, providing functional context for the microbial transcriptional changes described below. We therefore focus on microbial DEGs associated with oxidation–reduction processes and the transformation of chemically complex organic compounds rather than treating the transcriptome alone as direct proof of NAFC degradation.

**Figure 5:**
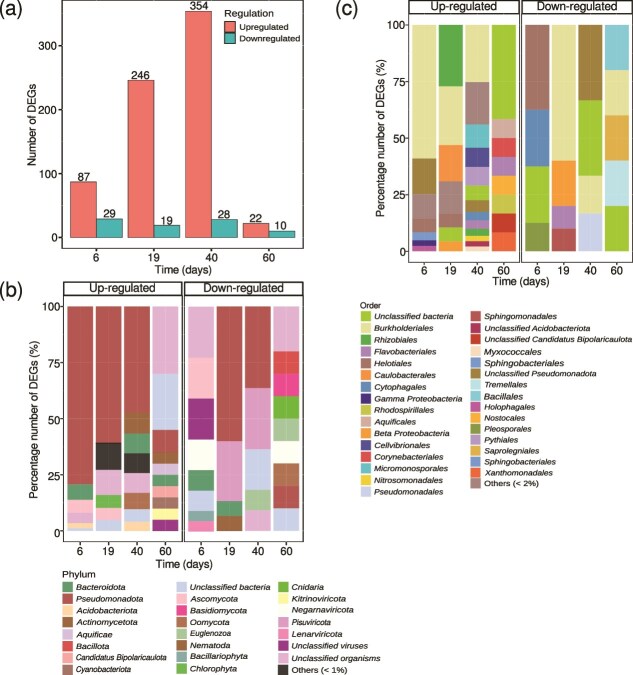
Differentially expressed microbial genes (DEGs) in *T. latifolia* roots and their taxonomic affiliations. (a) Number of DEGs at each time point. Taxonomic affiliations of DEGs at the (b) phylum and (c) order levels; low-abundance groups were collapsed into the “Others” category within the plot. Comparisons were performed between OSPW and ROW at matched sampling times. No DEGs were detected at D0, the pretreatment baseline.

The comparison between OSPW and ROW led to identification of 795 DEGs, with 89% being up-regulated (Fig. 5a; Table S8). The number of DEGs peaked at D40 (354) before sharply declining to D60 (22), coinciding with reduced NAFC concentrations in Balaberda *et al*. [14]. No DEGs were detected at D0, before refilling with distinct water types (ROW or OSPW), which was expected as all mesocosms had been filled with ROW up to that point. Most up-regulated genes were from *Pseudomonadota* (79.3% at D6, declining to 10% at D60), with the *Burkholderiales* and *Rhizobiales* orders as predominant contributors (Fig. 5b and c; Tables S9 and S10). Down-regulated genes were few and taxonomically diverse even among low-abundance orders. Enriched functional categories included translation, ribosomal structure, and stress-related pathways (Fig. S1; Table S11), suggesting protective responses to NAFC toxicity [74].

**Figure 6:**
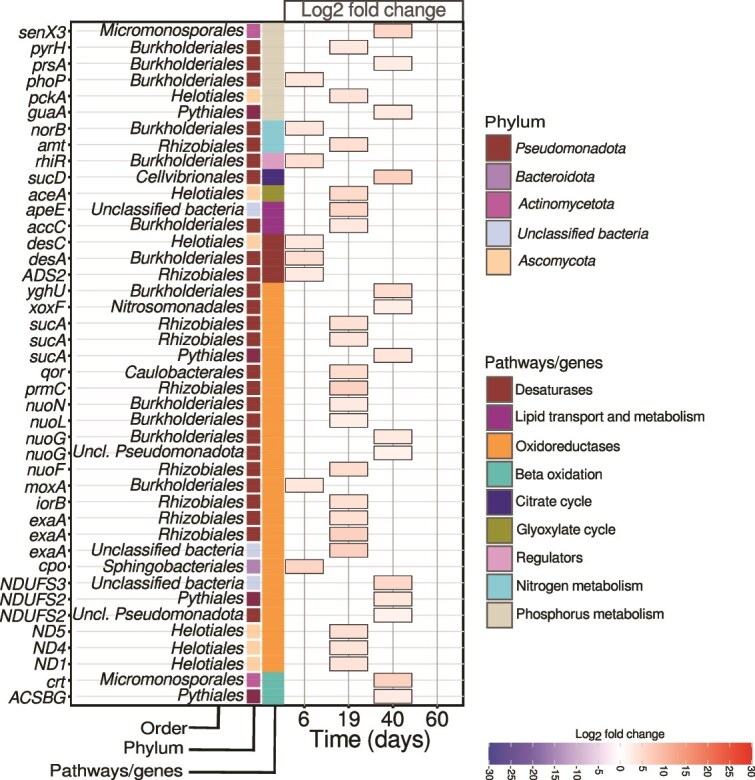
Differentially expressed genes (DEGs) with plausible relevance to naphthenic acid fraction compound transformation or to the metabolism of structurally related hydrocarbons. Comparisons were performed at matched sampling times between oil-sands process–affected water (OSPW) and reverse osmosis water (ROW) treatments. There were no identified DEGs at time 0 before adding OSPW; 6–60 represent sampling days after adding OSPW or refilling with ROW.

Focusing on NAFC-relevant metabolism, 42 up-regulated genes were associated with xenobiotic breakdown across D6, D19, and D40 (Fig. 6; Table S12), including genes involved in oxidoreduction, lipid metabolism, and nutrient (N, P, S) cycling [4, 74]. Most of these up-regulated genes were oxidoreductases or transport/metabolism-related enzymes. However, the 18 previously identified NAFC-degradation genes were not found among the up-regulated genes, but we identified specific oxidoreductase genes as reported by Reis *et al*. [4]. These oxidoreductases included NADH–ubiquinone/quinone oxidoreductases (ND1/4/5, NDUFS2/5 and *nuoFGLN*) for xenobiotic detoxification and free radical reduction [63, 75]; *cpo* (nonheme chloroperoxidase) for cleaving polymer chains [76]; *exaA* (alcohol dehydrogenase)—involved in chloroalkane/chloroalkene degradation [77]; *iorB* (isoquinoline 1-oxidoreductase) for targeting nitrogen-containing heterocycles [78]; *moxA* (manganese oxidase) for oxidizing a variety of phenolic model substrates [79, 80]; *prmC* (propane 2-monooxygenase) for degrading propane, acetone, and other volatiles [81, 82]; *qor* (NAD(P)H dehydrogenase [quinone]) for reducing and detoxifying quinone and various organics [63, 75]; *sucA* (2-oxoglutarate dehydrogenase) for catalysing the oxidative decarboxylation of 2-oxo acids to the corresponding acyl-CoA [83]; *xoxF* (lanthanide-dependent methanol dehydrogenase) for oxidizing alcohols and aldehydes [84]; and *yghU* (GSH-dependent oxidoreductase) for degrading xenobiotics, including lignin, atrazine, and dichloromethane [85]. Additionally, we identified some DEGs as desaturases (*ADS2, desA,* and *desC*), which catalyze C–C bond dehydrogenation [86], along with genes involved in lipid metabolism and nitrogen/phosphorus cycling. The genes found here were assigned to *Rhizobiales, Burkholderiales* (*Pseudomonadota*), and *Helotiales* (*Ascomycota*). Together, these results point to a broad oxidative and catabolic response to OSPW rather than a transcriptomic signature that can be assigned uniquely to NAFC degradation.

### Previously reported naphthenic acid fraction compound–associated genes in the present metatranscriptome

In our microbial differential analysis, the 18 genes previously linked to NAFC degradation [3] were not detected among the DEGs. This is likely because our comparison focused on OSPW versus ROW root samples, rather than the conventional planted versus unplanted rhizosphere mesocosms used in prior studies. Nevertheless, we identified 2488 sequences across all time points corresponding to previously characterized NAFC-degradation genes, confirming their active expression (Fig. S2; Table S13A). Gene expression peaked at D6 in both OSPW and ROW (sampled after refilling with OSPW or ROW) before declining by D60. The slight peak may be rapid transcriptional response to a slight environmental perturbation.

These genes are mostly involved in beta-oxidation pathways. This pattern is consistent with an early response to more bioavailable organic substrates, followed by reduced transcriptional activity as the labile compounds were depleted [87]. Expression was slightly higher after adding OSPW, indicating stimulation by OSPW exposure. Majority of expressed genes were taxonomically assigned to *Pseudomonadota*, which increased after adding or refilling at D6 and declined thereafter (Fig. S3a; Table S13B). This phylum was followed by the *Oomycota* phylum. At the order level, *Burkholderiales* dominated, followed by *Rhizobiales* and *Caulobacterales* (Fig. S4a; Table S13C), consistent with their known roles in NAFCs and organic hydrocarbon degradation [4].

### Abundance and taxonomic origins of hydrocarbon-degradation genes

To explore more NAFC-relevant pathways, we profiled the expression of 37 marker genes from the CANT-HYD database [36], leveraging structural similarities between hydrocarbons and NAFCs. These genes encode enzymes involved in anaerobic and aerobic degradation pathways of aliphatic and aromatic hydrocarbons. Of 37 marker genes, 25 transcripts were recovered, covering 132 sequences across all time points. Transcript abundance generally declined from D6 to D60, except for increases in *prmA* and *tmoA* in ROW (Fig. S5; Table S14A), which encode propane and toluene/benzene monooxygenases essential for oxidation of propane [88] and toluene [89], respectively. These genes were most abundant at D0 before refilling with OSPW, with other hydrocarbon-degradation genes dominating later, indicating a temporal shift in the hydrocarbon-related transcript pool over time. The same pattern was also observed in hydrocarbon aerobic degradation genes using HADEG (Fig. S6a–c; Table S15A–C), a manually curated database containing sequences of experimentally validated proteins and genes [37], which shares 27 genes with CANT-HYD database. CANT-HYD genes were mainly assigned to *Pseudomonadota, Actinomycetota*, and *Ascomycota* (Fig. S3b; Table S14B), with *Rhizobiales* and *Burkholderiales* as the largest contributors at the order level (Fig. S4b; Table S14C). Similarly, HADEG-derived genes followed similar taxonomic patterns (Fig. S3c; Table S15D), with varying contributions from *Burkholderiales, Rhizobiales*, and *Caulobacterales* (Fig. S4c; Table S15E). These orders are among the key taxa reported to possess genes for NAFC degradation [4].

### Abundance and taxonomic origins of nutrient cycling genes

We profiled genes that could be linked to anaerobic NAFC degradation, including those linked to nitrogen, sulfur, phosphorus, and methanogenic pathways [90, 91]. In general, there were no consistent temporal patterns in genes involved in N, S, or P metabolism across systems (Figs S7–S10; Tables S16–S19A and B). However, OSPW demonstrated a rise in sulfur-related genes at D6 (Fig. S8; Table S17A and B). These genes were primarily assigned to *Pseudomonadota, Bacteroidota, Ascomycota*, and *Oomycota* (Fig. S11A–D; Tables S16C–S19C), with *Burkholderiales* and *Rhizobiales* consistently dominating among orders (Fig. S12; Tables S16D–S19D). Both groups are well known for nitrate reduction, denitrification, sulfur cycling, and phosphorus mineralization [92–95], supporting their potential role in transforming heteroatom-containing organic constituents in OSPW. Methanogenesis was unlikely, as key *mcrABCDG* genes were absent (Fig. S10; Table S19); instead, expressed genes were linked to methylotrophy rather than methane production [96], with contributions from *Rhizobiales, Burkholderiales*, and other methylotrophic taxa.

## Conclusion

Our findings show coordinated plant and microbial transcriptional responses in *T. latifolia–*planted mesocosms previously shown to remove NAFCs from OSPW. The active microbiome was initially dominated by *Pseudomonadota*, especially *Burkholderiales*, and then shifted over time together with broad changes in functional gene expression. In parallel, *T. latifolia* roots up-regulated genes associated with oxidoreduction, transport, and conjugation, consistent with xenobiotic stress responses. These data help explain the stronger NAFC attenuation previously observed in planted mesocosms, but they do not by themselves demonstrate direct NAFC degradation by specific taxa or demonstrate plant-microbe synergy. Because OSPW is chemically complex, the transcriptional patterns observed here likely reflect combined responses to NAFCs and other dissolved constituents. Future work should pair transcriptomics with time-resolved chemistry, isolate-based validation, and plant-only or microbe-only controls to directly test the mechanisms suggested here.

## Supplementary Material

Supplementary_materials_ycag152

## Data Availability

The metatranscriptomic fastq files were deposited under the NCBI BioProject PRJNA1358281 (see Table S20). For reproducibility, reusability, and transparency, we have also made available the co-assembled library (doi: https://doi.org/10.6084/m9.figshare.30667601). All scripts used for data analysis and figure generation are publicly available on Zenodo (https://doi.org/10.5281/zenodo.17553524) and GitHub (https://github.com/Julius-Nweze/Exp3_differential_expression_analysis).
